# Increased neurokinin-1 receptor availability in the amygdala in social anxiety disorder: a positron emission tomography study with [^11^C]GR205171

**DOI:** 10.1038/tp.2015.92

**Published:** 2015-07-07

**Authors:** A Frick, F Ahs, C Linnman, M Jonasson, L Appel, M Lubberink, B Långström, M Fredrikson, T Furmark

**Affiliations:** 1 Department of Psychology, Uppsala University, Uppsala, Sweden; 2 Department of Clinical Neuroscience, Karolinska Institutet, Stockholm, Sweden; 3 Center for Pain and the Brain, Department of Anesthesiology, Perioperative and Pain Medicine, Boston Children's Hospital, Harvard Medical School, Boston, MA, USA; 4 Department of Nuclear Medicine and PET, Uppsala University, Uppsala, Sweden; 5 Department of Chemistry, Uppsala University, Uppsala, Sweden

## Abstract

The neurokinin-1 (NK1) receptor is abundantly expressed in the fear circuitry of the brain, including the amygdala, where it modulates stress and anxiety. Despite its proposed involvement in psychopathology, only a few studies of NK1 receptor availability in human subjects with anxiety disorders exist. Here, we compared NK1 receptor availability in patients with social anxiety disorder (SAD; *n*=17) and healthy controls (*n*=17) using positron emission tomography and the radiotracer [^11^C]GR205171. The Patlak Graphical plot using a cerebellar reference region was used to model the influx parameter, *K*_i_ measuring NK1 receptor availability. Voxel-wise statistical parametric mapping analyses revealed increased NK1 receptor availability specifically in the right amygdala in SAD patients relative to controls. Thus, we demonstrate that exaggerated social anxiety is related to enhanced NK1 receptor availability in the amygdala. This finding supports the contribution of NK1 receptors not only in animal models of stress and anxiety but also in humans with anxiety disorders.

## Introduction

While activity patterns in the neural fear circuitry, including the amygdala, hippocampus, anterior cingulate cortex and insula are altered in anxiety conditions,^1^ the neurochemical underpinnings of these disabling and common^2^ psychiatric disorders are not fully understood. The neuropeptide substance P (SP) and its preferred neurokinin-1 (NK1) receptor^3, 4^ are abundantly expressed in the brain's fear circuit,^5^ and have been suggested to have a role in anxiety disorders.^6, 7, 8^ The evidence mainly stems from animal and pharmacological intervention studies showing that the SP/NK1 system in the amygdala modulates stress and anxiety.^6, 9^ For example, stress increases the release of SP in the amygdala in rats.^9^ Consistently, in humans, patients with posttraumatic stress disorder (PTSD) exhibit elevated cerebrospinal fluid SP concentrations that are further heightened by symptom provocation.^10^ Also, exposure to phobic stimuli reduces the binding of the radiolabeled NK1 receptor antagonist GR205171 in the amygdala in patients with specific phobia, consistent with displacement of the tracer by stress-induced endogenous SP release.^11^

Moreover, in animals, administration of SP into the amygdala has anxiogenic effects,^9, 12^ whereas pharmacological blockage of the NK1 receptor is associated with decreased anxiety-like behavior.^6, 9^ Similarly, in healthy human subjects, NK1 antagonists have anxiolytic effects.^13^ Also, in patients with social anxiety disorder (SAD), treatment with the selective NK1 antagonist GR205171 alleviates clinical symptoms and attenuates anxiety-induced regional cerebral blood flow in the amygdala.^14^ However, it should be noted that treatment findings from clinical trials of NK1 antagonists for psychiatric disorders are mixed.^15, 16, 17, 18, 19, 20^ Collectively, findings both in animals and humans support that SP, acting through NK1 receptors, is anxiogenic.

Although there is ample evidence that the SP/NK1 system is involved in anxiety, not much is known about NK1 receptor availability in patients with anxiety disorders. To the best of our knowledge, only one positron emission tomography (PET) imaging study on this topic has been published to date, in which Fujimura and colleagues reported a widespread decrease of NK1 receptor availability in patients with panic disorder as compared with healthy control individuals.^21^

SAD, one of the most common anxiety disorders with a life-time prevalence of 10–15%,^2^ is associated with a hyperactive fear circuit, most consistently the amygdala, during anxiety provocation^22^ and emotional perception.^23^ Given the role of the SP/NK1 system in stress and anxiety, and because treatment with NK1 receptor antagonists has shown promising initial results in SAD, including attenuation of stress-related amygdala activity,^14^ it could be hypothesized that this disorder is associated with altered NK1 receptor availability particularly in the amygdala. The aim of the present study was therefore to examine NK1 receptor availability in SAD patients as compared with healthy controls (HCs) using PET and the highly selective NK1 receptor antagonist radiotracer [^11^C]GR205171.^24^

## Materials and methods

### Participants

Eighteen SAD patients and 18 age- and sex-matched HCs were included. Due to technical problems, data from one participant in each group could not be analyzed, leaving 17 participants with SAD (nine women; mean±s.d. age: 30.9±7.3 years) and 17 HCs (nine women; mean±s.d. age: 34.6±9.8 years). Participants were recruited through newspaper advertising. Initial screening consisted of social anxiety questionnaires and a brief telephone interview including self-report of alcohol consumption during the last month. The anxiety disorder questions from the Structured Clinical Interview for DSM-IV disorders^25^ was thereafter administered by a clinical psychologist. Duration of SAD symptoms was assessed through self-report. In addition, to exclude other serious psychiatric disorders, a psychiatrist administered the Mini International Neuropsychiatric Interview^26^ and a medical examination was conducted.

All patients met the DSM-IV^27^ criteria for SAD and exhibited marked public speaking anxiety. Social anxiety symptom severity was measured using the Liebowitz Social Anxiety Scale Self-Report version (LSAS-SR).^28^ Six of the SAD patients fulfilled criteria for a comorbid psychiatric disorder, four for general anxiety disorder, one for specific phobia and one for both generalized anxiety disorder and specific phobia, and one patient had a history of major depressive disorder. Four patients had a history of treatment with selective serotonin re-uptake inhibitors, and two with propranolol. One patient still used propranolol occasionally before entering the study, while all other patients were free of psychotropic medication for at least 6 months. None of the HCs met criteria for previous or current psychiatric disorder.

Main exclusion criteria were current primary psychiatric disorder other than SAD, alcohol/drug abuse, neurological disorder, somatic disease, ongoing treatment for SAD or treatment terminated within 6 months, chronic use of prescription medication, left handedness, previous PET examination, family history of cancer and for women also pregnancy and menopause.

### PET image acquisition

PET image acquisition was performed using an ECAT Exact HR+ PET scanner (Siemens/CTI, Knoxville, TN, USA) with an axial field of view of 155 mm. Subjects fasted 3 h and refrained from alcohol, caffeine and tobacco 12 h before the PET investigation, assessed by self-report.

Subjects were positioned supine in the PET scanner with their heads lightly fixated and a venous catheter was inserted. A 10-min transmission scan was performed using three retractable germanium (^68^Ge) rotating line sources. After the transmission scan, [^11^C]GR205171 was injected intravenously as a fast bolus simultaneously with the start of the emission scan. [^11^C]GR205171 is a selective NK1 receptor antagonist with subnanomolar affinity to the receptor as well as rapid uptake in the brain.^24, 29, 30^ Subjects rested during image acquisition. Data were acquired in three-dimensional mode and consisted of 17 frames (4 × 60 s, 3 × 120 s, 10 × 300 s) with a total duration of 60 min. The SAD group received on average 378.6 (SD: 25.0) MBq, equal to 5.4 (SD: 1.1) MBq kg^−1^ body weight, and the HC group received 398.1 (SD: 11.3) MBq, equal to 5.6 (SD: 1.1) MBq kg^−1^ body weight. In addition, a [^15^O]water PET scan used for spatial normalization was acquired (three frames × 30 s) with the administration of ~10 MBq kg^−1^ body weight. Both SAD patients and HC participants underwent the same investigations.

### Image analysis

Parametric images showing influx rate *K*_i_ (ml cm^−3^ min^−1^) of [^11^C]GR205171 for each voxel, that is, an index of NK1 receptor availability, were calculated using a modified Patlak Graphical plot with cerebellum as reference region^11, 31, 32^ using the time interval of 30–60 min. The cerebellum was chosen as the reference region as it displays a paucity of NK1 receptors.^17, 24, 33^ Definition of cerebellum was performed using the [^15^O]water PET scan of each participant and PVElab software,^34^ an observer-independent approach for automatic generation of volumes of interest.

Each individual's [^11^C]GR205171 *K*_i_ image was co-registered to their [^15^O]water summation image using affine transformation. The [^15^O]water summation image was then normalized to the PET template from Statistical Parametric Mapping 8 (Wellcome Department of Cognitive Neurology, University College London, www.fil.ion.ucl.ac.uk), and the calculated transformation parameters applied to the [^11^C]GR205171 *K*_i_ image, resulting in [^11^C]GR205171 *K*_i_ images normalized to the Montreal Neurological Institute standard space with isotropic 2 × 2 × 2 mm^3^ voxels. The Montreal Neurological Institute-normalized [^11^C]GR205171 *K*_i_ images were subsequently smoothed with a 12-mm isotropic Gaussian kernel.

### Data transformation

Alcohol consumption was recoded into consumed centiliters of 40% alcohol last month. One beer was counted as 6.25 cl of 40% alcohol and one bottle of wine as 22.5 cl. Two SAD patients did not provide data that could be recoded, that is, they did not quantify their alcohol consumption.

### Statistical analysis

Anatomical regions of interest (ROIs) were chosen *a priori* on the basis of earlier neuroimaging findings in SAD and included nodes in the brain fear circuitry: the amygdala, hippocampus, insular cortex and anterior cingulate cortex.^1^ ROIs were defined using the Automated Anatomical Labeling library from the Wake Forest University Pickatlas.^35^ In addition, a whole-brain exploratory analysis was performed.

Statistical analyses were conducted using Statistical Parametric Mapping 8. Group differences in NK1 receptor availability between SAD patients and HC participants were assessed using two-sample *t*-tests. To explore the relationship between social anxiety symptom severity and NK1 receptor availability within the SAD group, parametric [^11^C]GR205171 *K*_i_ images were entered into a regression model with LSAS-SR total score as predictor. Age and sex were entered as covariates in all the analyses, as there are known effects of age and sex on NK1 receptor availability.^36, 37^ For ROI analyses, the statistical threshold for significance was set to *P*<0.05 family-wise error (FWE) corrected for multiple comparisons within the ROIs, and for the exploratory whole-brain analysis the statistical threshold was set at *P*<0.001 uncorrected to not miss small differences in NK1 receptor availability. Follow-up analyses of ROIs displaying significant group differences were conducted to calculate the mean percent difference between the SAD and HC groups using the mean NK1 receptor availability within the whole anatomically defined ROIs.

Behavioral data and participant characteristics were analyzed using R 3.1.0 (R Foundation for Statistical Computing, Vienna, Austria).

### Ethical statement

The study was approved by the Uppsala University Medical Faculty Ethical Review Board and the Radiation Ethics Committee at Uppsala University Hospital. All study participants gave written informed consent before the study start and were reimbursed for their participation.

## Results

LSAS-SR scores were significantly higher (*t*(32)=13.37, *P*<0.001) in the SAD patients (mean±s.d.: 80.6±20.6) than in the HC group (mean±s.d.: 6.6±5.0). Mean duration of SAD symptoms was 19.4 (9.4) years. The groups did not differ in educational level (*χ*^2^(1)=0.14, *P*=0.71) or alcohol consumption (SAD: mean±s.d. 64.3±86.2 cl; HC: 54.4±54.9; *t*(30)=0.40, *P*=0.70).

**Figure 1:**
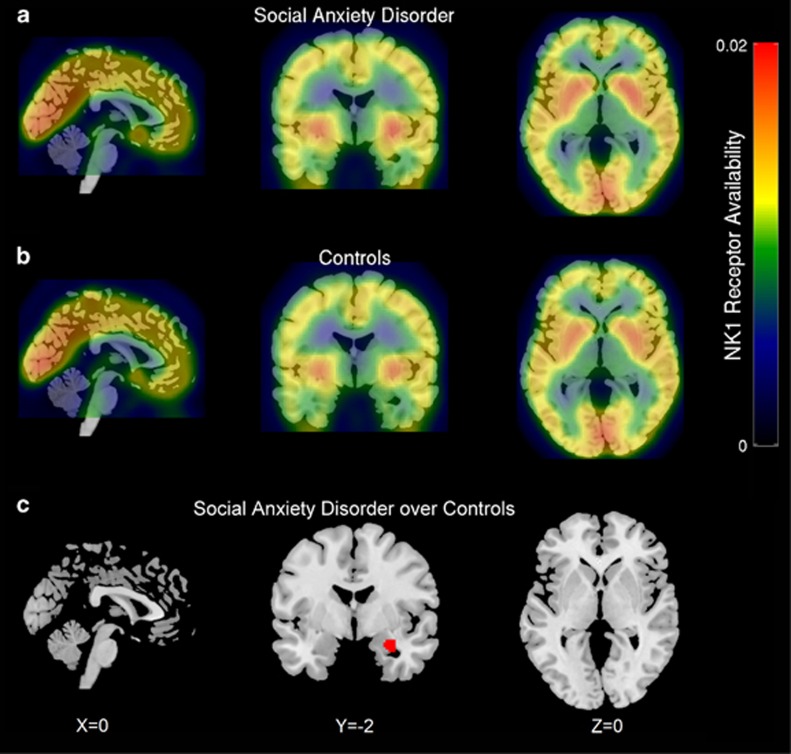
Parametric [^11^C]GR205171 *K*_i_ images showing mean neurokinin-1 (NK1) receptor availability in patients with (**a**) social anxiety disorder and (**b**) healthy controls. The color bar indicates [^11^C]GR205171 *K*_i_ values. (**c**) Patients with social anxiety disorder showed increased NK1 receptor availability in the amygdala. Voxels within the amygdala were thresholded at *P*<0.05, family-wise error corrected for multiple comparisons. Mean parametric images of [^11^C]GR205171 *K*_i_ and the statistical maps from the group comparison were overlaid on standard MRI images. All rows depict slices at MNI coordinate (0, −2, 0). MNI, Montreal Neurological Institute; MRI, magnetic resonance imaging.

**Figure 2:**
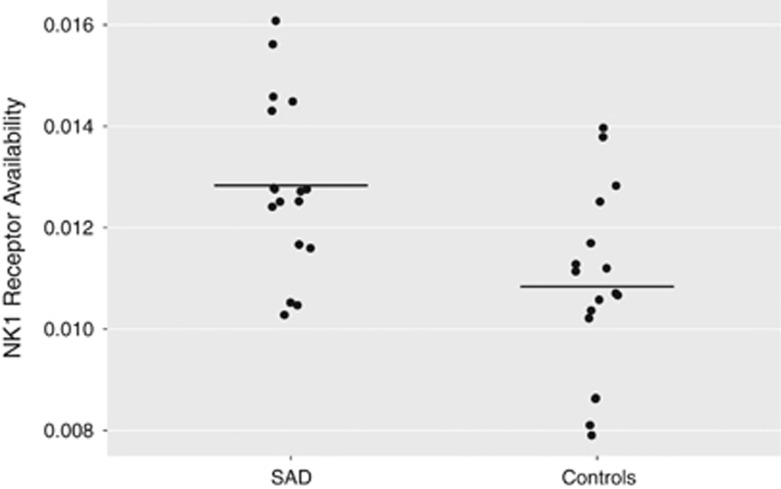
Neurokinin-1 (NK1) receptor availability ([^11^C]GR205171 *K*_i_) in the right amygdala in patients with social anxiety disorder (SAD) and healthy controls. Black horizontal lines denote group averages.

Statistical parametric mapping within the *a priori* ROIs (the amygdala, hippocampus, insular cortex and anterior cingulate cortex) revealed higher NK1 receptor availability in the right amygdala only (Montreal Neurological Institute x, y, z: 28, −2, −20; *Z*=3.79, *P*_FWE_=0.004; 496 mm^3^) in SAD patients as compared with HC individuals (see Figure 1). Mean regional NK1 receptor availability in the right amygdala ROI was 18.5% higher in SAD patients (mean±s.d.: 0.0128±0.0017) than in HCs (mean±s.d.: 0.0108±0.0018) (*t*(32)=3.294, *P*=0.002; see Figure 2). The exploratory whole-brain analysis revealed no additional areas except for the amygdala cluster present also in the ROI analyses. There were no significant associations between social anxiety symptom severity and NK1 receptor availability, or between duration of SAD symptoms and NK1 receptor availability. No significant changes in NK1 receptor availability were noted when comparing patients with generalized SAD to non-generalized SAD. Removing patients with psychiatric comorbidity or history of psychotropic medication did not alter the results, as the right amygdala uptake remained highly significantly different between patients and controls (Montreal Neurological Institute x, y, z: 28, −4, −20; *Z*=3.16, *P*_FWE_=0.03; 104 mm^3^).

## Discussion

In this PET study, we demonstrate increased amygdala NK1 receptor availability in SAD patients relative to controls, consistent with a role for NK1 receptors in human anxiety disorders as suggested by previous animal and human research.^6, 9, 11, 14, 21^

The present finding of enhanced NK1 receptor availability in the amygdala is paralleled by previous reports of heightened amygdala reactivity in SAD during emotional challenges,^22, 23^ in accordance with the notion that amygdala NK1 receptors are involved in stress-induced reactions.^11^ Consistently, NK1 receptor antagonism in SAD is associated with reduced state anxiety and attenuated amygdala responses during stressful public speaking.^14^ The association between fear-related neuronal activity and the SP/NK1 system is further strengthened by preclinical research showing that stress-induced activity in fear-relevant regions is mediated by NK1 receptors, and that NK1 receptor antagonism attenuates this activity.^8^ It is also noteworthy that a positive feedback mechanism is involved in stress-related SP release such that NK1 activation triggers SP release during stress, leading to activation of additional neurokinin receptors where SP binds with low affinity.^38^ The heightened resting state NK1 receptor availability in SAD may thus reflect an increased capacity for stress-related upregulation of SP release, and thereby also exaggerated amygdala activity, consistent with increased SP release and amygdala activation in response to symptom provocation in patients with PTSD^10, 39^ and specific phobia.^11, 40^ Blocking NK1 receptors in patients with comorbid PTSD and alcoholism increases activity in the ventromedial prefrontal cortex,^41^ an area involved in emotion regulation through its projections to the amygdala^42, 43^ often reported to be hypoactive in PTSD.^44^ Intriguingly, single administration of the NK1 antagonist aprepitant to healthy participants enhances anterior cingulate cortex and amygdala activity to positive stimuli, but does not reduce fear-related neural activity,^45^ possibly due to the need for prolonged NK1 blockage or stronger negative stimuli. Further studies are necessary to determine whether the association between activity in the SP/NK1 system and fear-related amygdala responsivity reflects a pathophysiological pathway linking neurochemical alterations to exaggerated neural reactivity.

Our findings of elevated NK1 receptor availability in the right amygdala of patients with SAD suggest a role for the SP/NK1 system in human anxiety disorders, but stand in contrast to the findings of widespread reduction in NK1 receptor availability in patients with panic disorder.^21^ Similar discrepancies have been reported for the serotonin transporter (SERT), that is, SAD is associated with increased SERT availability,^46^ whereas in panic disorder, males, but not females, exhibit elevated SERT availability.^47, 48^ Moreover, it should be noted that findings from clinical trials of NK1 receptor antagonists for mood and anxiety disorders have been mixed. Initial positive findings^14, 20^ have been difficult to replicate in phase III studies.^16, 17^ A proposed reason for this discrepancy is the lack of sufficient receptor occupancy in phase III trials.^49, 50^ Furthermore, NK1 receptor blockage may be selectively effective for symptoms during high stress levels such as symptom provocation not ordinarily assessed in clinical trials. Indeed, in a previous report from our lab, NK1 receptor antagonism reduced state anxiety during stressful public speaking but not overall social anxiety symptoms in patients with SAD relative to placebo,^14^ and in a recent trial of GR205171 for PTSD, only hyperarousal symptoms were significantly improved.^19^ Interestingly, NK1 receptor antagonists have also shown potential for treatment of alcohol- and drug-related disorders,^41, 51, 52^ that are often comorbid with mood and anxiety disorders.^53, 54^

Even though the spatial resolution of PET is limited, the location of the peak voxel, according to the detailed atlas of Mai *et al.,*^55^ was in the basolateral amygdala in which NK1 receptor previously have been linked to anxiety-like behavior.^56^ As SAD is arguably associated with heightened stress levels,^57^ increased NK1 receptor availability in socially anxious individuals is consistent with animal research on long-term upregulation of SP receptors in the amygdala following prolonged restraint stress.^58^ Indeed, it has been suggested that stress-induced effects on SP release and NK1 receptor expression could be modulated by stressor duration and intensity.^6, 9^ We, therefore, argue that the increased binding of resting [^11^C]GR205171 in SAD patients is a marker of enhanced NK1 receptor availability in the amygdala following prolonged stress, rather than reflecting lower endogenous SP concentrations. However, it remains to be investigated whether enhanced NK1 receptor availability is a risk factor for developing SAD or a consequence of the disorder.

Furthermore, the SP/NK1 system is frequently co-expressed and interacts with the serotonin system,^59^ suggesting that the present findings may be linked to compromised brain serotonin function in SAD and other anxiety disorders.^60^ Indeed, NK1 receptor antagonists enhance brain serotonergic cell activity,^61^ whereas blocking serotonin re-uptake decreases SP concentrations in fear-related brain regions including the amygdala.^62^ The NK1 receptor also has a role in regulating oxytocin secretion.^63^ Accordingly, NK1 receptor antagonists,^14^ selective serotonin re-uptake inhibitors^14, 64^ and oxytocin^65^ attenuate amygdala responses to emotional challenge in SAD, suggesting a common final pathway for these potentially anxiolytic agents.

Some limitations of the study deserve mentioning. First, there was restricted power, due to relatively few participants, to find a relationship between the SP/NK1 system and clinical symptoms at the behavioral level, although the number of subjects included is a normal sample size in PET trials. Second, our statistical thresholds may be perceived as liberal, but it should be noted that the increase in amygdala NK1 receptor availability survived stringent Bonferroni correction for number of ROIs analyzed. Also, since the exploratory analysis revealed no other areas that differed between groups, the amygdala difference is specific. Unfortunately, genotype information was not available, precluding genetic association analysis, e.g., the recently reported influence of TACR1 genotype on amygdala NK1 receptor availability.^66^

In conclusion, we demonstrate increased NK1 receptor availability in the amygdala of SAD patients. Enhanced SP/NK1 neurotransmission may exaggerate fear-related amygdala activity and our results may thus help explain previous reports of enhanced amygdala responses to socioemotional stimuli in SAD^23^ and attenuated amygdala reactivity, as well as anxiety reduction, following NK1 antagonism.^14^ The findings support involvement of the SP/NK1 system not only in animal models of stress and anxiety^6^ but also in humans with anxiety disorders.
